# Enteral versus parenteral nutrition in the conservative treatment of upper gastrointestinal fistula after surgery: a multicenter, randomized, parallel-group, open-label, phase III study (NUTRILEAK study)

**DOI:** 10.1186/s13063-020-04366-3

**Published:** 2020-06-02

**Authors:** Caroline Gronnier, Cécile Chambrier, Alain Duhamel, Benoît Dervaux, Denis Collet, Delphine Vaudoyer, Jean-Marc Régimbeau, Jacques Jougon, Jérémie Théréaux, Gil Lebreton, Julie Veziant, Alain Valverde, Pablo Ortega-Deballon, François Pattou, Muriel Mathonnet, Julie Perinel, Laura Beyer-Berjot, David Fuks, Philippe Rouanet, Jérémie H. Lefevre, Pierre Cattan, Sophie Deguelte, Bernard Meunier, Jean-Jacques Tuech, Patrick Pessaux, Nicolas Carrere, Ephrem Salame, Eleonor Benaim, Bertrand Dousset, Simon Msika, Christophe Mariette, Guillaume Piessen, on behalf of FRENCH association

**Affiliations:** 1 Department of Visceral Surgery, Centre Médico-chirurgical Magellan, Pessac, France; 2 Department of Nutrition and Intestinal Rehabilitation, Lyon Sud University Hospital, Pierre-Bénite, France; 3 Pôle de Santé Publique, Department of Biostatistic, University Hospital of Lille, Lille, France; 4 Maison Régionale de la Recherche Clinique, Hospitalière et Universitaire - CHRU de Lille, Lille, France; 5 Department of Visceral Surgery, Lyon Sud University Hospital, Pierre-Bénite, France; 6 Department of Visceral Surgery, Amiens University Hospital, Amiens, France; 7 Department of Thoracic Surgery, Centre Médico-chirurgical Magellan, Bordeaux University Hospital, Pessac, France; 8 Department of Visceral Surgery, Hospital Center Regional University, Brest, France; 9 Department of Visceral Surgery, Caen University Hospital, Caen, France; 10 Department of Visceral Surgery, Gabriel-Montpied Hospital, Clermont-Ferrand, France; 11 Department of Visceral Surgery, Diaconesses Hospital, Paris, France; 12 Department of Visceral Surgery, University Hospital Dijon Bourgogne, Dijon, France; 13 Department of Visceral and Endocrine Surgery, Huriez University Hospital, Lille, France; 14 Department of Visceral Surgery, Dupuytren University Hospital, Limoges, France; 15 Department of Visceral Surgery, Edouard Herriot University Hospital, Lyon, France; 16 Department of Visceral Surgery, Hôpital Nord, Marseille, France; 17 Department of Visceral Surgery, Institut Mutualiste Montsouris, Paris, France; 18 Department of Visceral Surgery, Institut Regional Cancer Montpellier, Montpellier, France; 19 Department of Visceral Surgery, Saint-Antoine University Hospital, Paris, France; 20 Department of Visceral Surgery, Saint-Louis University Hospital, Paris, France; 21 Department of Visceral Surgery, Robert Debré University Hospital, Reims, France; 22 Department of Visceral Surgery, Pontchaillou University Hospital, Rennes, France; 23 Department of Visceral Surgery, Rouen, France; 24 Department of Visceral Surgery, Nouvel Hôpital Civil, Strasbourg, France; 25 Department of Visceral Surgery, Purpan University Hospital, Toulouse, France; 26 Department of Visceral Surgery, Tours, France; 27 Department of Visceral Surgery, Gustave Roussy Institute, Villejuif, France; 28 Department of Visceral Surgery, Cochin Hospital, Paris, France; 29 Department of Visceral Surgery, Bichat Hospital, Paris, France; 30 Department of Digestive and Oncological Surgery, Lille University Hospital, Lille, France

**Keywords:** Enteral nutrition, Parenteral nutrition, Conservative treatment, Upper gastrointestinal fistula, Randomized controlled trial

## Abstract

**Background:**

Postoperative upper gastrointestinal fistula (PUGIF) is a devastating complication, leading to high mortality (reaching up to 80%), increased length of hospital stay, reduced health-related quality of life and increased health costs. Nutritional support is a key component of therapy in such cases, which is related to the high prevalence of malnutrition. In the prophylactic setting, enteral nutrition (EN) is associated with a shorter hospital stay, a lower incidence of severe infectious complications, lower severity of complications and decreased cost compared to total parenteral nutrition (TPN) following major upper gastrointestinal (GI) surgery. There is little evidence available for the curative setting after fistula occurrence. We hypothesize that EN increases the 30-day fistula closure rate in PUGIF, allowing better health-related quality of life without increasing the morbidity or mortality.

**Methods/design:**

The NUTRILEAK trial is a multicenter, randomized, parallel-group, open-label phase III trial to assess the efficacy of EN (the experimental group) compared with TPN (the control group) in patients with PUGIF. The primary objective of the study is to compare EN versus TPN in the treatment of PUGIF (after esophagogastric resection including bariatric surgery, duodenojejunal resection or pancreatic resection with digestive tract violation) in terms of the 30-day fistula closure rate. Secondary objectives are to evaluate the 6-month postrandomization fistula closure rate, time of first fistula closure (in days), the medical- and surgical treatment-related complication rate at 6 months after randomization, the fistula-related complication rate at 6 months after randomization, the type and severity of early (30 days after randomization) and late fistula-related complications (over 30 days after randomization), 30-day and 6-month postrandomization mortality rate, nutritional status at day 30, day 60, day 90 and day 180 postrandomization, the mean length of hospital stay, the patient’s health-related quality of life (by self-assessment questionnaire), oral feeding time and direct costs of treatment. A total of 321 patients will be enrolled.

**Discussion:**

The two nutritional supports are already used in daily practice, but most surgeons are reluctant to use the enteral route in case of PUGIF. This study will be the first randomized trial testing the role of EN versus TPN in PUGIF.

**Trial registration:**

ClinicalTrials.gov: NCT03742752. Registered on 14 November 2018.

## Background

The incidence of clinically significant postoperative upper gastrointestinal fistula (PUGIF) surgery is approximately 4%–20%, and the associated mortality can be as high as 80% [1]. Various clinical presentations are described that can endanger the patient’s life. It is important to start explorations without delay when the diagnosis of PUGIF is made as delayed management could lead to a devastating prognosis. Optimal communication between the teams is mandatory for successful treatment. When the clinical presentation is an important and acute sepsis the treatment should be surgical, but when the clinical presentation is less symptomatic or late the treatment should be more conservative with a watch-and-wait or endoscopic management [2, 3]. The diagnosis should be made with the help of computed tomography (CT) scans with oral contrast and low-insufflation early endoscopy. The principles of treatment include transfer to the intensive care unit, optimal perfusion, intensive physiotherapy, antifungal and antibiotic treatment, and eventually drainage and collection or fistula closure (when possible) in the absence of ischemia [2].

Some promising endoscopic techniques in the treatment of PUGIF have been reported (such as over-the-scope (OVESCO)-clip®), mainly in bariatric surgery and often in case reports [4]. The results give information on feasibility but are too weak to give information on efficiency. Large defects cannot be treated with hemostatic clips [2, 4, 5]. The fistula output can be reduced by somatostatin analogs [3, 6].

Several prognostic factors of PUGIF have been identified [7], such as high output, high concentration of toxic bile acids and active digestive enzymes, a fistula tract longer than 2 cm, elevated postoperative blood glycemia [8] and malnutrition with serum albumin <30 g/l [3].

Nutritional support is a key component of PUGIF management, related to the high prevalence of malnutrition and nil-per-mouth requirements for fistula treatment [9, 10]. Therefore, despite fasting, nutritional support is mandatory, and both enteral nutrition (EN) downstream of the site of leakage (via a feeding jejunostomy or a nasojejunal feeding tube placed radiologically or endoscopically) [11] and parenteral nutrition are possible and currently used. However, the role of EN in maintaining the small intestinal structure and function and in improving postoperative outcomes is well established. Enteral nutrients maintain the structural function that are compromised by fasting and parenteral nutrition [12, 13].

In the prophylactic setting (i.e., in a population without fistula but at the risk of developing one) a published systematic literature review [1] based on seven randomized trials showed that EN is associated with shorter hospital stay, lower incidence of severe infectious complications [14], lower severity of complications and decreased costs compared to total parenteral nutrition (TPN) following major upper gastrointestinal (GI) surgery [1, 15].

In the curative setting (after fistula occurrence) there is evidence available. Only one randomized clinical trial has suggested the superiority of EN over TPN after pancreatic surgery, with an increase in the 30-day fistula closure rate from 37% in the TPN group to 60% in the EN group [16]. This trial only included pancreatic fistula and did not include all PUGIFs that can also occur after esophagogastric resection, including bariatric surgery, duodenojejunal resection or pancreatic resection with digestive tract violation, although the concept of enteral nutritional support is highly relevant in all these situations. Even if EN seems to be promising, the potential risk of increasing the leakage output related to a reflux of nutritional liquid and/or to activate digestive enzymes, consequently reducing the probability of fistula closure rate or increasing the delay of fistula closure, may explain why surgeons are usually reluctant to provide EN [4]. A few, small randomized studies suggested the feasibility of EN in 47 patients with upper GI fistula [17] for the treatment of esophagojejunal fistula after total gastrectomy in gastric cancer patients [18] and after PUGIF following sleeve gastrectomy [19, 20], but to date no randomized study has been designed to test the superiority of EN versus TPN in PUGIF.

Our study aim is thus to demonstrate the superiority of EN over TPN in accelerating fistula healing after upper GI surgery.

## Methods/design

### Protocol overview

The NUTRILEAK trial is a multicenter, randomized, parallel-group, open-label, phase III study to assess the efficacy of EN compared to TPN in patients with PUGIF. After informed consent, patients will be randomized in a 2:1 ratio to the EN treatment arm and the TPN comparator arm. Patients will be randomized to receive EN through jejunostomy or nasojejunal tube, or to receive TPN through central venous access, a peripherally inserted central catheter line, totally implantable venous access or any other approved TPN device.

Any other surgical or endoscopic procedures aiming at directly closing the defect will not be allowed (including surgical closure and endoscopic clip, prosthesis or glue) throughout the whole study period. These measures have not been scientifically demonstrated to be efficient in this situation at this time and could be a confounding factor regarding our primary objective.

By contrast, surgical, radiological or endoscopic fistula drainage will be allowed throughout the whole study period. Any interruption of more than 24 h in the treatment determined by randomization (EN or TPN) will be reported, with the cause, duration and solutions given (i.e., the treatment provided or a switch to TPN in the EN group). During hospitalization, patients will be evaluated daily until fistula closure, with any fistula- or protocol treatment-related complication viewed through physical examination and, if required, through routine laboratory tests and/or imaging according to the usual practice of each center.

**Fig. 1:**
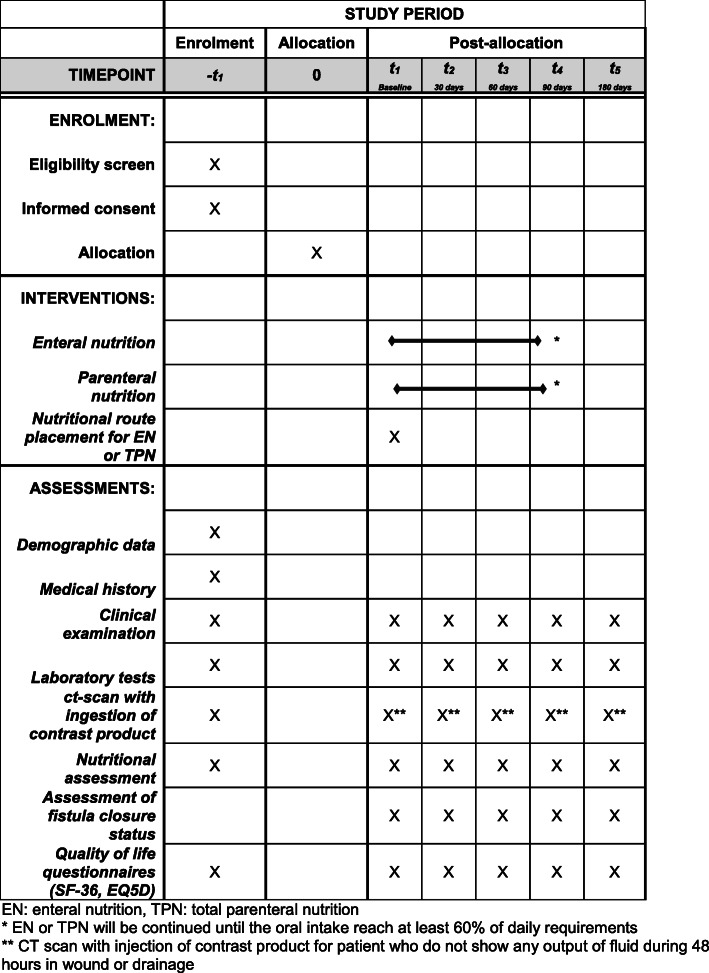
Schedule of the trial interventions and assessments. *Enteral nutrition (EN) or total parenteral nutrition (TPN) will be continued until the oral intake reaches at least 60% of daily requirements. **Computed tomography (CT) scan with contrast injection for patient who do not show any output of fluid during 48 h in wound or drainage. EQ5D EuroQol five dimensions, SF-36 36-item Short Form

The present study protocol was written in compliance with the Standard Protocol Items: Recommendations for Interventional Trials (SPIRIT) 2013 [21]. A completed SPIRIT checklist is available as Additional file 1, and the schedule of this study is presented in Fig. 1.

**Fig. 2:**
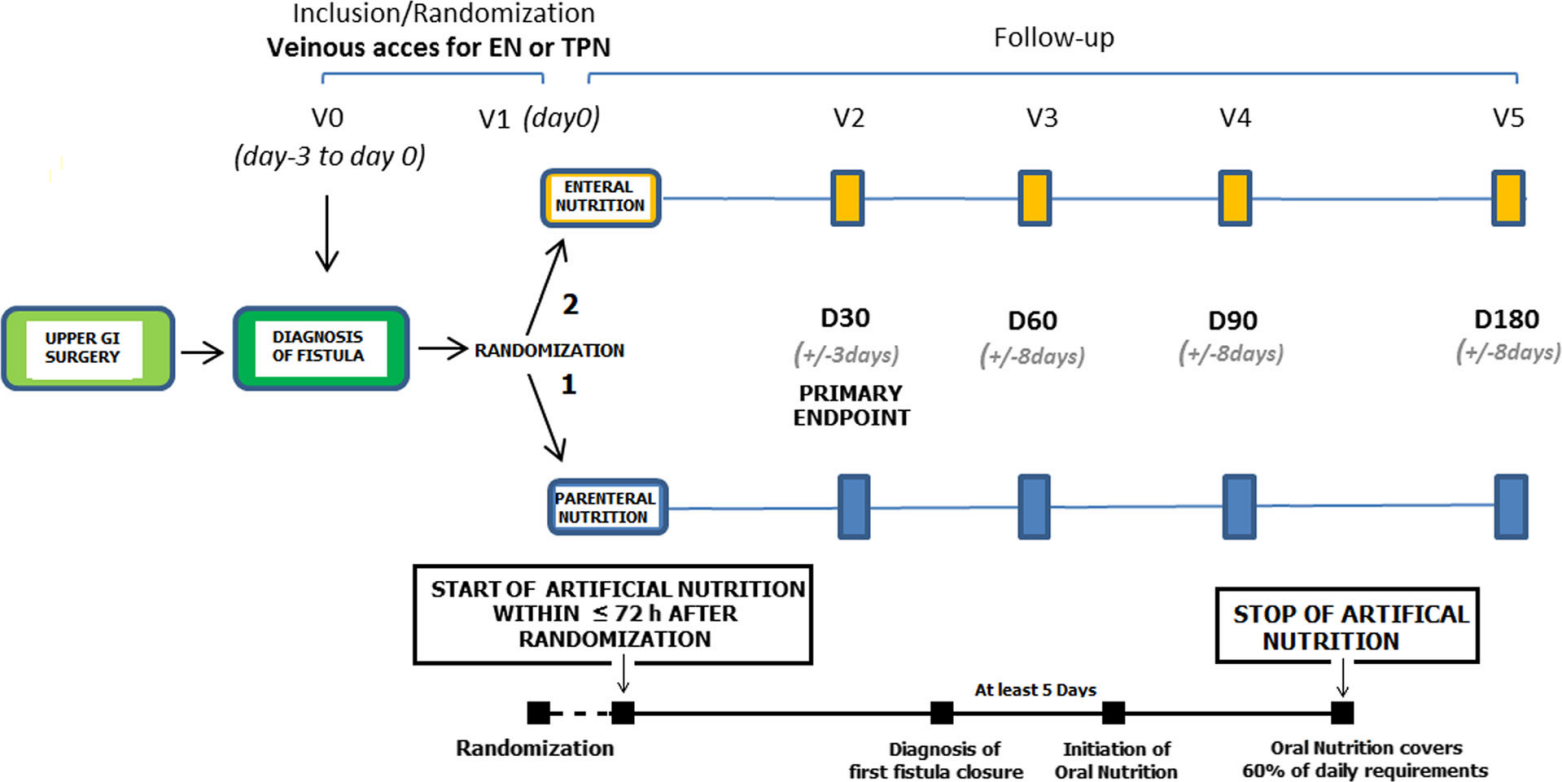
Flowchart of the trial. D day, EN enteral nutrition, GI gastrointestinal, TPN total parenteral nutrition, V visit

The study flowchart is shown in Fig. 2.

### Objectives

The primary objective is to demonstrate the superiority of EN over TPN in the treatment of PUGIF after esophagogastric resection including bariatric surgery, duodenojejunal resection or pancreatic resection with digestive tract violation in terms of the 30-day fistula closure rate.

Secondary objectives are the assessment of: 1) 6-month postrandomization fistula closure rate; 2) time of first fistula closure (in days); 3) medical and surgical treatment-related complication rate (EN or TPN) at 6 months after randomization; 4) fistula-related complication rate at 6 months after randomization; 5) type and severity of early (30 days after randomization) and late (over 30 days after randomization) fistula-related complications; 6) 30-day and 6-month postrandomization mortality; 7) nutritional status at day 30, day 60, day 90 and day 180 postrandomization; 8) mean length of hospital stay; 9) patient health-related quality of life (by self-assessment questionnaire); 10) time for oral feeding; and 11) direct costs of treatment.

### Inclusion criteria

All patients diagnosed with a postoperative digestive fistula in the recruiting centers will be screened for eligibility to participate in the study. Inclusion criteria are patients who: 1) are older than 18 years of age; 2) signed the written informed consent form (Additional file 2); 3) underwent upper GI surgery for benign or malignant disease (i.e., esophagogastric resection including bariatric surgery, duodenojejunal resection or pancreatic resection with digestive tract violation); 4) have had the diagnosis of an active postoperative digestive fistula untreated or persisting after failure of a dedicated surgical or endoscopic procedure to close the fistula (the fistula should have been diagnosed less than 72 h before randomization and confirmed by at least two criteria among the following: clinical symptoms, CT scan/ultrasonic imaging/endoscopic diagnosis, biologic/bacteriology diagnosis on fluid output, intraoperative diagnosis at time of reoperation); 5) have the indication of nil-per-mouth or just clear liquids for comfort; 6) require an artificial nutritional support; 7) have an American Society of Anesthesiologists (ASA) score of 1, 2 or 3; 8) have a life expectancy longer than 6 months; 9) have no history of allergy or product intolerance to the nutritional product used in the study; 10) have ongoing healthcare insurance; and 11) are able to understand the information letter.

### Exclusion criteria

Patients with any of the following criteria will not be included in the study: 1) scheduled surgical or endoscopic treatment with the aim of closing the fistula (suture, prosthesis, clip or glue); in case of treatment failure, patients are eligible to participate to the study; endoscopic or surgical drainage are not exclusion criteria (meaning that drainage is authorized only before randomization); 2) patient diagnosed with an isolated pancreatic fistula (without digestive content) after a pancreatic resection without digestive tract violation; 3) history of current severe uncontrolled cardiovascular, pulmonary, renal or liver failure; 4) presence of a severe and evolutive life-threatening pathology; 5) uncontrolled sepsis/situation related to the fistula (including, but not limited to, abscess, bleeding, fistula with the trachea or the aorta); 6) requirement of a nutritional support combining both the enteral and parenteral routes together; 7) peritoneal carcinomatosis or distant metastasis; 8) pregnant and/or lactating women; 9) freedom privacy; and 10) patient currently participating or having participated in another interventional clinical trial related to nutritional support or fistula management during 30 days prior to the beginning of the study (note that participation in a prior clinical trial not related to nutritional support or fistula management does not exclude the patient from participation).

### End points

The primary end point is the fistula closure rate at 30 days after randomization. Fistula closure will be defined as no output fluid for 48 h in the wound or drainage and absence of any fluid collection on imaging (CT scan with contrast injection). The secondary end points are: 1) 6-month fistula closure rate; 2) time of first fistula closure, defined as time in days from randomization to first fistula closure within 6 months after randomization; 3) medical and surgical treatment-related complication rate at 6 months, including complications related to the nutritional support such as tube -related complications (dislodgment, infection, occlusion), venous catheter -related complications (thrombosis, infection), or any other nutritional route-related complications; 4) fistula-related complication rate at 6 months; 5) type and severity of early (before 30 days after randomization) and late (over 30 days after randomization) complications according to the Dindo–Clavien classification [22]; 6) mortality within 30 days and within 6 months after randomization; 7) nutritional status will be evaluated at day 30, day 60, day 90 and day 180 postrandomization based on weight, serum albumin and prealbumin concentration, C-reactive protein and grip test (muscular strength); 8) length of hospital stay in a health care structure (including home hospitalization) based on the cumulative number of days of hospitalization during the whole study period (from randomization until the end of the study); 9) patient health-related quality of life score based on the 36-item Short Form and EuroQoL five-dimensions questionnaires at inclusion and at days 30, 60, 90 and 180; 10) time from randomization to the oral diet covering at least 60% of the patient’s daily requirement ; 11) direct economic costs of therapy from a societal perspective, including the costs of hospitalization (inpatient and home settings), nutritional products, and early and late complications occurring during follow-up.

### Randomization

Patients will be randomized at inclusion during hospitalization after verification of their suitability for inclusion. Patients will be randomized using the Clinsight system (ENNOV). Randomizing two cases for one control has been chosen based on the positive results of the study by Klek et al. exhibiting a higher 30-day fistula closure rate in the EN group for patients with pancreatic fistula, which has a similar context [16]. In addition, European guidelines are in favor of EN for patients needing artificial nutrition with a grade A level of evidence (without the context of PUGIF where nothing has been demonstrated to date). A dynamic randomization procedure by minimization will be performed to achieve a balance between the following prognostic factors: the type of fistula (high- versus low-output, where a high-output fistula is defined as effluent greater than 200 ml/24 h), malignant/nonmalignant disease, and somatostatin analog use. The variable of treatment center will also be considered in the minimization procedure. Taking into account the fistula outflow, stratification on the surgical procedure/organ will not be included in the minimization procedure since the two are strongly linked.

### Treatment methods

Each nutritional support will be planned to provide a similar amount of calories and proteins. According to the French guidelines on perioperative nutrition [23] the amount of calories will be 30–35 kcal/kg/day including proteins. The protein or amino acid intake will represent 18–20% of caloric intake: 1.35–1.5 g/kg/day (i.e., nitrogen at 0.21–0.24 g/kg/day).

EN or TPN will start in the first 72 h following randomization with the need for tube or catheter placement (if not already placed and according to randomization arm) in the meantime.

EN can be delivered through a jejunostomy or a nasojejunal tube. A polymeric hypercaloric hyperprotidic product without immunonutrients will be chosen. The nutritional product will be the one usually used in each center. EN will be started slowly (20 ml/h) with a progressive increase in the infusion rate every day according to the tolerance of the patient. The expected infusion rate to cover nutritional needs should be obtained in a week. If the tolerance of EN does not allow the energy and protein requirements to be met at the end of the first week it will be necessary to start a complementary parenteral nutrition to make up the nutritional requirements. EN will be infused at a continuous rate via an enteral pump. In the case of obstruction or failing of the enteral tube, a feeding tube should be replaced immediately with the agreement of the surgeon. If it is impossible to replace an enteral tube, parenteral nutrition should be started on the same day.

TPN will be delivered through a central venous access, a peripherally inserted central catheter line or a totally implantable venous access port or any other approved TPN device. The parenteral nutritional product will be chosen according to the patient’s nutritional requirements as defined in the study and according to the usual care of each center (industrial or compounding bag). However, it is recommended to avoid a parenteral formulation containing a long-chain triglyceride lipid emulsion and to add intravenous glutamine (Dipeptiven®) in the TPN [24]. If Dipeptiven® is added to parenteral nutrition (recommended dose of 0.3–0.5 g/kg/day of dipeptide), the amount of amino acids (or nitrogen) will be included in the calculated amount of protein intake. Parenteral nutrition will include electrolytes, vitamins and trace elements every day. Serum levels of phosphate should be assessed every 72 h. TPN should be infused over 24 h with a pump. In case of catheter obstruction or bacteremia related to the catheter, the central venous catheter will be replaced and complications will be treated according to guidelines [25, 26].

Patients will have appropriate care according to local procedures to avoid complications with the feeding tube or enteral venous catheter.

For all patients, glycemia will be checked regularly. In case of hyperglycemia (>10 mmol/l or >1.8 g/l), glycemia should be maintained between 7.8 and 10 mmol/l (1.4 to 1.8 g/l) with the use of insulin.

Nil-per-mouth (except a maximum of 500 ml/day of clear liquids for comfort) will be required during fistula treatment and until at least 5 days after fistula closure. After this, oral alimentation will be progressively introduced under the supervision of a nutritionist using a previously known energetic value, and the proportion of oral alimentation ingested will be monitored daily.

### Data collection and follow-up

Follow-up of the patients will be at 30, 60, 90 and 180 days after randomization. The follow-up protocol includes a clinical examination (weight, temperature, arterial blood pressure), assessment of the status of fistula closure, paraclinic examination (CT scan with injection and ingestion of contrast product) for patients who do not show any output of fluid during 48 h in the wound or drainage, assessment of time to first fistula closure through the autoevaluation of fistula-associated symptoms questionnaire, laboratory tests (blood cell count, hemoglobin, white blood cell count with neutrophils, lymphocytes, monocytes, eosinophils, basophils, platelets, hematocrit, red blood cell count, aspartate transaminase, alanine aminotransaminase, alkaline phosphatase, γ-glutamyltransferase, total bilirubin, creatinine, C-reactive protein, serum albumin and prealbumin concentration, total protein, sodium, potassium, chloride, urea, glucose), nutritional assessment (weight, total protein, serum albumin and prealbumin concentration), a grip test for muscular strength measurement, assessment of the World Health Organization Performance Status, and quality of life questionnaires (36-item Short Form and EuroQoL five-dimensions questionnaire).

The study is planned to last 42 months, with a 36-month inclusion period and a 6-month follow-up period. The results of the primary end point will be available 30 days after the end of the inclusion period (3 years).

### Participating centers

To prevent institutional bias, the centers participating in this trial are experienced in upper GI surgery. In this study, 27 French centers will participate: Lille University Hospital (two departments), Bordeaux University Hospital (two departments), Lyon University Hospital (two centers), Amiens University Hospital, Brest University Hospital, Caen University Hospital, Clermont-Ferrand University Hospital, Diaconesses Hospital, Dijon University Hospital, Limoges University Hospital, North University Hospital of Marseille, Institut Mutualiste Montsouris, Montpellier University Hospital, Saint-Antoine University Hospital, Saint- Louis University Hospital, Bichat University Hospital, Cochin University Hospital, Institut Gustave Roussy, Reims University Hospital, Rennes University Hospital, Rouen University Hospital, Strasbourg University Hospital, Toulouse University Hospital and Tours University Hospital.

### Statistical evaluation and sample size

The hypothesis of this phase III study is that the use of EN will improve the fistula closure rate at 30 days after randomization. According to Klek et al. [16], the 30-day fistula closure rate is expected to be 35% in the TPN group and 55% in the EN group. According to Rutegård et al. [27], we expect 10% mortality within 30 days. Considering a 35% fistula closure rate in the TPN group and an expected 55% rate in the EN group (absolute difference of 20%, relative risk 1.57), a two-sided test, a type I error of 0.05, a 90% power, an allocation rate of 2:1 for the EN group and the TPN group, respectively, and taking into account 30-day mortality of 10%, 214 patients are needed in the EN group and 107 in the TPN group, leading to a total number of 321 patients to be recruited.

The intention-to-treat (ITT) population will comprise all randomized patients, whether or not they satisfy the eligibility criteria and irrespective of the study treatment actually received. Unless otherwise indicated, all efficacy and safety analyses (including the primary outcome) will be conducted on the ITT population.

The per-protocol (PP) population will consist of all ITT patients who complied with the protocol requirements. Compliance with protocol requirements will be addressed through the review of protocol deviations/violations at the time of a blind data review meeting, just prior to database lock. Any significant issues may warrant patient exclusion of all or part of their assessment data. The PP population will be applied only for the primary outcome and will be considered as a secondary analysis. For example, if an EN subject was intolerant for 48 h, and then switched to TPN for support, they will be analyzed in the EN group in the ITT analysis and the TPN group in the PP analyses.

No interim analysis is planned.

Statistical analyses will be independently performed by the Biostatistics Department of the University of Lille under the responsibility of AD. Data will be analyzed using SAS software (SAS Institute Inc., Cary, NC, USA) and all statistical tests will be performed with a two-sided alpha risk of 0.05. A detailed statistical analysis plan will be written and finalized prior to the database lock. The data analysts will be blinded to the treatment arm. Any deviation from the protocol-specified analysis will be documented within a protocol amendment or statistical analysis plan, as appropriate, and described within the clinical study report.

Patient accountability will be summarized by treatment group and overall for all randomized patients. In addition, patient accountability information for screen-failure patients will be collected and reported. The number of patients randomized will be summarized along with the number of patients within each patient population. In addition, the number of patients completing/not completing the study will be presented along with the primary reason for withdrawal from the study.

Deviations that warrant patient exclusion from the PP population will be determined just prior to database lock and documented within the relevant patient listing.

Some subgroup analyses, considered as exploratory, will be performed according to some well-known factors linked to the primary outcomes and considered in the randomization per minimization technique: 1) type of fistula (high- versus low-output fistula where high-output fistula is defined as effluent greater than 200 ml/24 h); 2) malignant/nonmalignant disease; and 3) somatostatin analog use or not.

Baseline characteristics will be described for each arm for the ITT population. Quantitative variables will be expressed as mean (standard deviation), median (interquartile range) and range. Qualitative variables will be expressed as frequencies and percentages. Normality of distributions will be assessed graphically and using the Shapiro–Wilk test.

### Medico-economic analysis

Considering the clinical design, a full economic evaluation will be performed taking into account both benefits and costs. The analysis will be conducted in concordance with Haute Autorité de Santé guidelines [28]. Cost-effectiveness analysis will be performed according to the ITT principle and from a societal perspective. A PP complementary analysis is planned as many patients are expected to switch from TPN to EN. The following costs will be considered in the economic analysis: 1) hospitalization (inpatient and home settings); 2) nutritional products; and 3) management of early (before 30 days after randomization) and late (over 30 days after randomization) complications occurring during follow-up.

The costs of hospitalization (inpatient) will be computed using the French hospital production costs study (Echelle Nationale des Couts à la Methodologie Commune Medecine Chirurgie Obstetrique). The average cost will be adjusted to the length of stay (secondary end point) and the number of days in intensive care units, which are known to be the main cost drivers. Home hospitalizations will be valued by reference to the French home hospitalization production costs study (Echelle nationale des Couts-Hospitalisation à Domicile). All hospitalizations will be recorded in the electronic case report form (gathering data on the hospital to which the patient was admitted, their main diagnosis, the date of admission and the date of discharge) at each scheduled clinical examination. Information on diagnosis-related groups (Groupe Homogène de Malades; inpatient setting) or management-related groups (Groupe Homogène de Prise en Charge; home care setting) will be requested at the end of the study by the study coordinator from hospitals to which patients were admitted. Nutritional products will be valued at their current price.

Quality-adjusted life years will be computed using the French value set by a linear interpolation between dates of measurement (inclusion and days 30, 60, 90 and 180) [29]. Considering follow-up, costs and quality-adjusted life years will not be discounted.

### Ethical approval

This study protocol was approved on 2 November 2018 by the national ethics board and written informed consent will be obtained from all participants in the trial by the study investigators in each center. The results will be presented at scientific meetings and published in periodicals.

### Confidentiality

Information about study subjects will be kept confidential. All data will be entered into a dedicated data management system and, as in all data document studies, subjects will be assigned an individual identifying code which does not contain any identifying information.

## Discussion

Despite considerable improvements in surgical techniques, PUGIF remains a worrying problem and there is an urgent requirement for improving outcomes after PUGIF normally associated with high mortality and morbidity. Nutritional support is mandatory to accelerate the healing of the fistula. However, the best route to deliver nutrition is still subject to debate in the literature. EN and TPN are already used in daily practice, but some surgeons are often reluctant to use EN in PUGIF.

Nutrients via the GI tract stimulate a complex response that has implications on body composition and immunologic integrity. The mechanisms include nonspecific luminal stimulation provided by nutrients, “functional workload”, potential stimulation of pancreaticobiliary secretions, secretion of humoral mediators, and induction of intestinal hyperemia [12, 13].

EN has been shown to be efficient in the prophylactic setting [1, 15], leading to a high probability of efficiency in the curative setting when the fistula is in place. In a well-designed randomized trial, EN was associated with significantly higher closure rates and shorter closure time for postoperative pancreatic fistula [16]. EN was identified as an independent factor significantly associated with fistula closure (odds ratio 6.136, 95% confidence interval 1.204–41.623; *P* = 0.043).

The transversal approach in this trial, with the inclusion of various upper GI surgeries, will help to validate the concept of the use of EN after fistula across different surgical subspecialties. The trial results could modify national and international guidelines and practices worldwide, offering a high level of evidence.

To conclude, in the NUTRILEAK study we aim to test the hypothesis of the superiority of EN over TPN in PUGIF in a large, multicentered, phase III, prospective controlled, open-label trial. This trial will also assess patient quality of life and medico-economic effects of the different treatment strategies.

## Trial status

This is protocol version 2.0 (1 February 2019). The trial is registered at ClinicalTrials.gov with the identifier NCT03742752. This trial is currently ongoing. The recruitment of subjects began in February 2019 is expected to finish in February 2022.

## Supplementary information

**Additional file 1.** SPIRIT 2013 checklist: recommended items to address in a clinical trial protocol and related documents.

**Additional file 2.** Consent form.

## Data Availability

The datasets used and/or analyzed during the current study will be available from the corresponding author on reasonable request.

## Abbreviations

CT: Computed tomography
EN: Enteral nutrition
GI: Gastrointestinal
ITT: Intention-to-treat
PP: Per-protocol
PUGIF: Postoperative upper gastrointestinal fistula
SPIRIT: Standard Protocol Items: Recommendations for Interventional Trials
TPN: Total parenteral nutrition
