# Supplementary material for: Enteral versus parenteral nutrition in the conservative treatment of upper gastrointestinal fistula after surgery: a multicenter, randomized, parallel-group, open-label, phase III study (NUTRILEAK study)
Source: Trials. 2020 Jun 2;21:448. doi: 10.1186/s13063-020-04366-3 (PMC7265255; doi:10.1186/s13063-020-04366-3)
Supplement: Supplementary file 2 — Additional file 2. Consent form. [file 13063_2020_4366_MOESM2_ESM.docx]

**Biomedical Research Participation Consent Form for the research untitled:**

**“Enteral versus parenteral nutrition in the conservative treatment of gastrointestinal fistula after upper gastro-intestinal surgery: a prospective, open label, multicentric, randomized, controlled, phase III study (NUTRILEAK)”**

**Sponsor of the study: Coordinating investigator:**

Centre Hospitalier Universitaire de Lille Pr. Guillaume PIESSEN

2, Avenue Oscar LAMBRET General and Digestive Surgery Department

59037 Lille Cedex Hôpital Claude HURIEZ - CHU Lille

Rue POLONOWSKI - 59037 Lille Cedex

Tél. : 03 20 44 44 07

Fax : 03 20 44 51 43

I, the undersigned Mrs., Mr. (delete whichever does not apply).................................................................

freely and voluntarily agree to participate in the biomedical research untitled:

**“Enteral versus parenteral nutrition in the conservative treatment of gastrointestinal fistula after upper gastrointestinal surgery: a prospective, open-label, multicentric, randomized, controlled, phase III study** **(NUTRILEAK)”,**

whose sponsor for France is Lille University Hospital and which was proposed to me by Doctor/Professor (name, telephone number) ………………………………………………

It being understood that:

1. I confirm that I have read and understood the briefing note for the NUTRILEAK study. I had time to read the information about the objectives, the duration, the effectiveness, the expected benefits and the possible side effects of the study. I was able to ask all the questions that I wanted to ask and I received answers to them in a satisfactory manner.
2. I understand that my participation is voluntary and that I am free to withdraw at any time without giving any reason, without my medical care or my rights being affected. I confirm that I belong to a social security scheme.
3. I agree that the data recorded in the course of this study may be computer-processed in a strictly anonymous manner. I have noted that the rights of access provided by the law of January 6, 1978, concerning IT, files and freedoms and by the European regulation on the protection of personal data (2016/679) (Articles 12 and following)^1^, may be exercised at any time to the doctor in charge of my care or to the Sponsor's Data Protection Officer (DPO), and that I will be able to exercise my right of rectification and of objection to this same doctor or the DPO, who will contact the research sponsor to assert my rights.
4. I understand that some of my medical information and personal data will be collected during the course of the study, and may be reviewed by the study investigators. I agree that persons appointed by the sponsor (doctors, clinical research assistants) may have access to my personal data and particularly to my medical data, for the purposes of monitoring the conduct of the trial.
5. Rights related to personal data are detailed in the information leaflet delivered to the participant.

Signed at …………………, on the…………………… Signed at …………………, on the………………

Signature of the participant Signature of the investigating doctor

*This form is produced in three copies, one of which is given to the person concerned. One copy will be kept by the investigator; the last will be kept by the sponsor, maintaining complete confidentiality, in accordance with the law.*
